# The Features of GGT in Patients with ATP8B1 or ABCB11 Deficiency Improve the Diagnostic Efficiency

**DOI:** 10.1371/journal.pone.0153114

**Published:** 2016-04-06

**Authors:** Neng-Li Wang, Li-Ting Li, Bing-Bing Wu, Jing-Yu Gong, Kuerbanjiang Abuduxikuer, Gang Li, Jian-She Wang

**Affiliations:** 1 Department of Pediatrics, Jinshan Hospital of Fudan University, Shanghai, China; 2 The Center for Pediatric Liver Diseases, Children’s Hospital of Fudan University, Shanghai, China; 3 The Molecular Genetic Diagnosis Center, Shanghai Key Lab of Birth Defects, Pediatrics Research Institute, Children’s Hospital of Fudan University, Shanghai, China; Texas A&M University, UNITED STATES

## Abstract

**Background and Aims:**

Genetic defects in *ATP8B1* or *ABCB11* account for the majority of cholestasis with low GGT. But the ranges for GGT in patients with ATP8B1 or ABCB11 deficiency are unclear. This study tried to unravel the features of GGT in these patients that improve diagnostic efficiency.

**Methods:**

This study enrolled 207 patients with chronic cholestasis who were ordered to test for *ATP8B1* and/or *ABCB11* from January 2012 to December 2015. Additional 17 patients with ATPB81 or ABCB11 deficiency diagnosed between January 2004 and December 2011 were also enrolled in this study. 600 population-matched children served as controls. Clinical data were obtained by retrospectively reviewing medical records.

**Results:**

A total of 26 patients were diagnosed with ATP8B1 deficiency and 30 patients were diagnosed with ABCB11 deficiency. GGT levels were similar between the two disorders at any observed month of age, but varied with age. The peak GGT value was <70U/L in the 2nd~6th month of life, <60U/L in the 7th~12th month and <50U/L beyond one year. GGT levels in patients with a genetic diagnosis were different from that in patients without a genetic diagnosis and controls. Larger ranges for GGT were found in patients without a genetic diagnosis. Some controls had GGT≥70U/L in the 2nd~6th month. Of the 207 patients, 39 (18.8%) obtained a genetic diagnosis. 111 patients met the ranges described above, including all the 39 patients with ATP8B1 or ABCB11 deficiency. The sensitivity was 100.0%. The rate of a positive molecular diagnosis increased to 35.1% (39/111 vs. 39/207, *X*^*2*^ = 10.363, *P* = 0.001). The remaining 96 patients exceeded the ranges described above and failed to receive a genetic diagnosis. These patients accounted for 43.8% of sequencing cost.

**Conclusions:**

GGT levels in patients with ATP8B1 or ABCB11 deficiency varied with age. The peak GGT value was <70U/L in the 2nd~6th month of life, <60U/L in the 7th~12th month and <50U/L beyond one year.

## Introduction

Cholestasis is one of the most common manifestations of liver disease in infancy and affects about 1 in 2500–5000 live births. A range of hepatobiliary disorders, that involve the process of bile acid biosynthesis, bile secretion and excretion, can result in cholestasis [1]. The etiology includes infectious, obstructive, metabolic, toxic as well as other rare causes [2]. Due to the advancement in molecular diagnoses, multiple specific genetic causes were identified, including citrin deficiency, Alagille syndrome, progressive familial intrahepatic cholestasis, inborn errors of bile acid synthesis, cystic fibrosis and so on [3].

In clinical practice, cholestasis is usually divided into two categories depending on serum GGT levels: those with elevated GGT and those with low/normal GGT [4]. The latter includes at least two distinct subtypes: ATP8B1 deficiency and ABCB11 deficiency. ATP8B1 deficiency is caused by mutations in *ATP8B1*, and can present as either progressive familial intrahepatic cholestasistype 1 (PFIC1) or benign recurrent intrahepatic cholestasis type 1 (BRIC1) [5,6]. ABCB11 deficiency is caused by mutations in *ABCB11*. The ABCB11 gene encodes bile salt export pump (BSEP) that is responsible for bile acid (BA) secretion [7]. Mutations in *ABCB11* can cause PFIC2 and BRIC2 [7,8]. Occasionally, BRIC can progress to PFIC [6,9]. Therefore, ATP8B1 or ABCB11 deficiency represents a spectrum of diseases, with PFIC as severe forms and BRIC as mild ones. Low serum GGT activity is the characteristic feature of these diseases.

GGT binds to the canalicular membrane by a glycosyl phosphatidyl inositol (GPI) anchor [10]. The detergent bile acid liberates GGT. In healthy infants, GGT activity varies with age [11–12]. The upper limit of GGT value could be 7 times of adult range in the first three months of life, then decreases gradually and reaches adult range at 6 months old [12–13]. It has been unclear whether GGT value in patients with *ATP8B1* or *ABCB11* deficiency varies as it does in healthy infants. The ranges for GGT in these patients were referred as normal, or low, or low for the degree of cholestasis [14–17]. The ambiguity might hinder the diagnosis. The aim of this study was to unravel the features of GGT in these patients that improve the efficiency of diagnosis.

## Methods

### Subjects

We retrospectively analyzed all patients who were ordered to screen for mutations in *ATP8B1* and/or *ABCB11* for chronic cholestasis between January 2012 and December 2015. 207 patients, including 112 boys and 95 girls, were enrolled in this study. Among them, 110 tested for both *ATP8B1* and *ABCB11*, 38 tested for *ATP8B1* and 59 tested for *ABCB11* only. The patients were referred to the Center for Pediatric Liver Disease of the Children’s Hospital of Fudan University and/or the Pediatrics Department of Jinshan Hospital of Fudan University. Following an extensive workup as described previously [13,18–20], other causes of chronic cholestasis were excluded, including infections, drug-induced, metabolic and inborn errors in bile acid synthesis. Cytomegalovirus (CMV) infection was defined as a positive urinary CMV-DNA or pp65 antigenemia or serum immunoglobulin M (IgM) [21–22], and not excluded because of its high prevalence in Chinese infants [23]. To expand the patient number, 17 patients with ATPB81 or ABCB11 deficiency diagnosed between January 2004 and December 2011 were also enrolled in this study. 36 patients with positive diagnoses were partly reported previously [18–20]. The patients were treated with ursodesoxycholic acid (UDCA) and fat-soluble vitamins. Cholestyramine was given if patients had intractable pruritus.

600 population-matched children severed as controls for analysis of GGT level variation in Chinese population. No liver disease history was found in these children. Liver function test was performed during airway infections course and revealed normal serum total bilirubin (TB) and normal alanine aminotransferase (ALT).

This study was approved by the ethics committee of the Children’s Hospital of Fudan University, and was dispensed from informed consent. Patients’ information was de-identified prior to analysis.

### Genetic analysis

Genetic analysis was performed in the Translational Center of Children’s Hospital of Fudan University. Genomic DNA was extracted from EDTA treated peripheral blood (Tiangen Biotech, Shanghai, China). All coding exons and splice junctions of *ATP8B1* (RefSeq NM_005603.3) and *ABCB11* (RefSeq NM_003742.2) were amplified by polymerase chain reaction (PCR) and directly sequenced as described previously [18–19]. Frameshift, nonsense, canonical splice site variations and previously reported mutations were considered deleterious. Missense variations predicted to be damaging were considered as potentially deleterious mutations. Deleterious and potentially deleterious mutations were confirmed by directly sequencing affected exons in both parents.

### Immunohistochemistry

Immunohistochemistry (IHC) was performed as reported previously [20]. The pathologists were blinded to genotyping results at assessment. Absent canalicular BSEP expression was defined as the typical IHC pattern for ABCB11 deficiency. Specific canalicular GGT expression, that was absent in centrilobular but preserved in periportal areas, was defined as the typical IHC pattern for ATP8B1 deficiency.

### Molecular diagnosis

Subjects were diagnosed with ATP8B1 or ABCB11 deficiency if deleterious or potentially deleterious mutations were detected in both alleles of *ATP8B1* or *ABCB11*, or in whom though only one mutant allele was detected in *ATP8B1* or *ABCB11* but decreased/absent GGT or BSEP expression was demonstrated respectively by IHC; otherwise the subjects were defined as patients lacking a genetic diagnosis.

### Clinical data and statistical analysis

The clinical data were obtained by reviewing medical records. GGT activities during disease course were used for statistical analysis, but those after liver transplantation, partial billiary diversion or liver failure were discarded. If GGT activities were tested several times within one month, the mean was used for comparison of GGT levels.

Statistic analysis was performed with the SPSS version 17.0 software (University of Chicago, Chicago, IL, United States). Data were expressed as median [P25, P75] for non-normality. Comparison of GGT levels between two groups was done by the nonparametric Mann-Whitney test. Comparison of GGT levels among three or more groups was performed by the nonparametric Kruskal-Wallis H test. The difference between two ratios was tested by Chi-square test. *P*<0.05 was considered significant.

## Results

### Molecular results

#### Mutations in *ATP8B1*

26 patients fulfilled the definition of ATP8B1 deficiency, including 1 heterozygote with weak canalicular GGT expression, 17 compound heterozygotes and 8 homozygotes (Table 1). 30 different mutations were identified, including eight novel mutations. 5 missense mutations were predicted to be damaging by Polyphen-2 and MutationTaster (S1 Table). c.1030-1G>A known to affect splicing. c.811A>T (p.R271X) and c.2013_2014delAA leaded to truncated protein.

**Table 1:** Clinic and genetic characteristics of 17 patients with ATP8B1 deficiency.

Patient no.	Sex/Age at onset	Symptoms^§^	Nucleotide change	Amino acid change	Origin	Ref
1	Male/1mo	J, P, H, S, FT	c.2081T>A/c.2081T>A	p.I694N/ p.I694N	F/M	Li et al [20]
2	Female/1mo	J, D, H	c.1367C>A/c.3292delG	p.T456K/p.V1098X	F/M	Li et al [20]
3	Female/4mo	J, P, H, S	c.886C>T/c.1675_1689delGTAAACGCTGCCAGG	p.R296C/p.V559_R563del	M/F	Li et al [20]
4	Male/14y	J, H	c.1982T>C/c.1982T>C	p.I661T/p.I661T	F/M	Present study
5	Female/1mo	J, P, H, S	c.625C>A+c.627+5G>T/c.2081T>A	p.P209T/p.I694N	M/F	Li et al [20]
6	Female/2mo	J, D, H	c.922G>A/c.2081T>A	p.G308S/p.I694N	F/M	Li et al [20]
7	Male/8y	J, D, P	**c.602G>A**/c.1587_1589delCTT	p.R201H/p.F527-	ND	Present study
8	Female/1mo	J, P, D, H	c.1367C>T/c.1587_1589delCTT	p.T456M/p.F527-	ND	Present study
9	Male/1mo	J, H	c.2821C>T/c.2821C>T	p.R941X/p.R941X	F/M	Li et al [20]
10	Female/1mo	J, D, H, S, FT	c.1336G>A/c.1587_1589delCTT	p.G446R/p.F527-	M/F	Li et al [20]
11	Female/4mo	J, P, H, S, FT	c.886C>T/c.2081T>A	p.R296C/p.I694N	ND	Li et al [20]
12	Male/1mo	J, H	c.2081T>A/c.2788C>T	p.I694N/p.R930X	ND	Li et al [20]
13	Male/15mo	J, P, H	c.920A>T/c.3401-2A>G	p.H307L/-	F/M	Li et al [20]
14	Male/1mo	J, H	**c.1030-1G>A**/**c.2013_2014delAA**	-/p.K672Vfs*17	ND	Present study
15	Female/3mo	J, P, D, H,S	c.1799G>A/c.1799G>A	p.R600Q/p.R600Q	F/M	Li et al [20]
16	Male/3mo	J, H	c.1799G>A/c.1799G>A	p.R600Q/p.R600Q	F/M	Present study
17	Female/1mo	J, H	c.614dupA/c.2532delT	p.N205KfsX2/p.K845RfsX36	ND	Present study
18	Male/2mo	J, H	c.625C>A+c.627+5G>T/ c.625C>A+c.627+5G>T	p.P209T/p.P209T	ND	Liu et al [19]
19	Male/1mo	J, P, H	c.1429+1G>A/c.1429+1G>A	-/-	ND	Liu et al [19]
20	Male/1mo	J, H	c.625C>A+c.627+5G>T/c.2081T>A	p.P209T/p.I694N	ND	Liu et al [19]
21	Female/1mo	J, D, H	c.625C>A+c.627+5G>T/ c.625C>A+c.627+5G>T	p.P209T/p.P209T	ND	Liu et al [19]
22	Male/1mo	J, H, S	c.614dupA/c.2532delT	p.N205KfsX2/p.K845RfsX36	ND	Liu et al [19]
23	Male/1mo	J, P, H, S	c.625C>A+c.627+5G>T/c.2854C>T	p.P209T/p.R952X	ND	Liu et al [19]
24	Male/2mo	J, H	**c.1264G>C**/**c.2734G>A**	p.D422H/p.G912R	F/M	Li et al [20]
25	Female/14y	J, P, D,H	**c.811A>T**/c.1882C>T	p.R271X/p.R628W	ND	Present study
26 ^‡^	Female/1mo	J, P, D, H, FT	**c.1661A>C**+**c.1741G>A**/-	p.D554A+p.E581K/-	M/-	Present study

J, jaundice; P, pruritus; D, diarrhea; H, hepatomegaly; S, splenomegaly; FT, failure to thrive; ND = not done; F, father; M, mother.

Novel mutations are shown in bold.

Patient 15 and 16, 17 and 22 are siblings.

^§^ Symptoms: the major symptoms when the patients were first referred to our hospital.

^‡^ Patient harboring the heterozygous *ATP8B1* mutation and having decreased GGT expression.

#### Mutations in *ABCB11*

30 patients were diagnosed with ABCB11 deficiency, including 4 homozygotes, 24 compound heterozygotes and 2 heterozygotes with absent BSEP expression (Table 2). Sequence analysis revealed 54 distinct mutations including 13 novel mutations. The 13 mutations included one nonsense mutation, two 1-base deletions leading to truncated protein, one splice site mutation known to affect splicing, and 9 missense mutations predicted to be damaging (S1 Table).

**Table 2:** Clinic and genetic characteristics of 18 patients with ABCB11 deficiency.

Patient no.	Sex/Age at onset	Symptoms^§^	Nucleotide change	Amino acid change	Origin	Ref
1	Female/1mo	J, H	c.1460G>A/c.3169C>T	p.R487H/p.R1057X	M/F	Present study
2	Female/1mo	J, P, H, S	c.1197+1G>T/c.1197+1G>T	-/-	F/M	Li et al [20]
3	Female/1mo	J, H, S, FT	c.2935A>G/c.3746T>G	p.N979D/p.L1249X	ND	Li et al [20]
4	Female/3mo	J, P, H, S	c.1415A>G/c.3392A>T	p.Y472C/p.D1131V	F/M	Present study
5	Male/1mo	J, H	c.634G>A+c.849A>C/c.1638G>T	p.A212T+p.E283D/p.Q546H	M/de novo	Li et al [20]
6	Female/1mo	J, H	c.1493T>C/c.1493T>C	p.I498T/p.I498T	F/M	Li et al [20]
7	Male/1mo	J, D, H, S	c.212T>A/c.677C>T	p.L71H/p.S226L	ND	Li et al [20]
8	Male/1mo	J, H	c.2782C>T/c.3593A>G	p.R928X/p.H1198R	M/F	Li et al [20]
9	Male/1mo	J, H	c.542G>T/c.1370_1372dupGTG	p.R181I/p.-458G	M/F	Li et al [20]
10	Female/1mo	J, H	c.3457C>T/c.3623A>G	p.R1153C/p.Y1208C	F/M	Li et al [20]
11	Female/5mo	J, P, H, S	**c.2474A>G**/**c.2623C>T**	p.E825G/p.Q875X	ND	Present study
12	Male/3y	J, P, H, S	c.1685G>A/**c.1847G>A**	p.G562S/p.R616H	F/M	Present study
13	Female/1mo	J, H, S	**c.113delA**/**c.2702G>T**	p.K38RfsX24/p.S901I	ND	Present study
14	Female/3y	J, P, H	**c.229C>G**/c.1880T>C	p.P77A/p.I627T	ND	Present study
15	Female/1mo	J, H, S	**c.2484_2488delA**/**c.2542G>A**	p.R830GfsX28 /p.D848N	ND	Present study
16	Female/2mo	J, P, H, S	**c.197G>A**/**c.555G>A**	p.S66N/p.M185I	ND	Present study
17	Female/1mo	J, P, H	**c.909-2A>G**/c.1550G>A	-/p.R517H	F/M	Present study
18	Female/1mo	J, P, H	c.1409G>A/**c.1762G>C**	p.R470Q/p.A588P	F/M	Present study
19	Female/1mo	J, H	**c.2603T>A**/c.3213+1G>T	p.V868D/-	M/F	Present study
20	Male/2mo	J, D, H, S	c.3457C>T/c.3623A>G	p.R1153C/p.Y1208C	F/M	Present study
21 ^‡^	Female/1mo	J, H, S	c.145C>T/-	p.Q49X/-	ND	Li et al [20]
22 ^‡^	Male/2mo	J, P, H, FT	c.612-2_4CTA>TT/-	-/-	M/-	Li et al [20]
23	Male/1mo	J, H, S	c.409G>A/c.2216delC	p.E137K/p.P740QfsX6	de novo/F	Li et al [20]
24	Male/1mo	J, H, S	c.1760C>G/c.3677G>C	p.S587X/p.R1226P	M/F	Li et al [20]
25	Male/2mo	J, D, H, FT	c.1583T>C/c.1583T>C	p.I528T/p.I528T	ND	Li et al [20]
26	Male/1mo	J, P, H	c.499G>A/c.499G>A	p.A167T/p.A167T	F/M	Liu et al [18]
27	Male/1mo	J, P, H, S	c.562G>T/c.2814+3A>T	p.G188W/-	ND	Liu et al [18]
28	Female/3mo	J, P, H	c.1496G>A/c.2606A>C	p.G499E/p.Q869P	F/M	Liu et al [18]
29	Male/1mo	J, P, H, S	c.319T>C/c.3172C>T	p.C107R/p.Q1058X	M/F	Liu et al [18]
30	Male/1mo	J, H, S	c.1243C>T/c.3875G>T	p.R415X/p.G1292V	F/M	Liu et al [18]

J, jaundice; P, pruritus; D, diarrhea; H, hepatomegaly; S, splenomegaly; FT, failure to thrive; ND = not done; F, father; M, mother.

Novel mutations are shown in bold.

Patient 10 and 20 are siblings.

^§^ Symptoms: the major symptoms when the patients were first referred to our hospital.

^‡^ Patient harboring the heterozygous *ABCB11* mutation and having absent BSEP expression.

### Laboratory evaluations

Only serum ALT and GGT activities in patients without a genetic diagnosis were different from that in the ATP8B1 deficiency and the ABCB11 deficiency simultaneously when liver function test results at presentation were compared among the three groups (Table 3). However, serum ALT activities were similar between patients with and without a genetic diagnosis (93.0[50.0, 187.5] vs. 100.0[41.7, 223.7], Z = -0.228, *P* = 0.819). GGT levels in patients without a genetic diagnosis were higher than that in patients with a genetic diagnosis (44.0[29.0, 65.5] vs. 29.0[19.0, 37.5], Z = -4.374, *P* = 0.000). In addition, no significant difference was found when the rates of CMV infection were compared between patients with and without a genetic diagnosis (17.9% vs. 23.8%, *X*^*2*^ = 0.858, *P* = 0.354).

**Table 3:** Biochemical features at presentation (median [P25, P75]).

	n	TB	ALT	ALP	GGT	BA	Albumin	Cholesterol	AFP
P _without_	168	150.2[94.6, 252.2]	93.0[50.0, 187.5]	542.5[372.7, 728.2]	44.0[29.0, 65.5]	171.8[101.1, 277.4]	40.2[36.6, 43.0]	4.5[3.3, 5.4]	2408.0[14.5, 91900.0]
P _ATP8B1_	26	195.8[161.0, 259.8]	46.0 [33.0, 87.0] *	588.5[396.0, 728.0]	27.0[18.0, 36.0] *	216.5[167.7, 287.9]	40.0[38.4, 41.5]	3.4[2.9, 4.2] *	33.7[4.5, 161.1] *
P _ABCB11_	30	124.7[101.3, 162.7]	186.0[109.5, 273.5] *^	386.5[250.5, 530.2] *^	32.0 [23.5, 38.5] *	294.1[205.2, 368.3] *^	43.9[40.0, 45.8] *^	4.4[3.0, 5.4] ^	1096.0[4.4, 23880.0] ^
*X*^*2*^		5.427	21.479	8.240	20.114	14.611	13.955	8.864	10.642
*P*		0.066	0.000	0.016	0.000	0.001	0.001	0.011	0.005

P_ATP8B1_, patients with ATP8B1 deficiency; P_ABCB11_, patients with ABCB11 deficiency; P_without_, patients without a genetic diagnosis; ALP, alkaline phosphatase; AFP, alfafetoprotein.

* compared with P _without_, *P*<0.05;

^ compared with P _ATP8B1_, *P*<0.05.

### GGT levels in patients with or without a genetic diagnosis and controls

A comparison of GGT levels between the ATP8B1 deficiency and the ABCB11 deficiency revealed that GGT levels were similar between the two disorders at any observed month of age (Table 4). However, GGT levels in patients with a genetic diagnosis were different from that in patients without a genetic diagnosis and controls. Compared to controls, patients with a genetic diagnosis, including the ATP8B1 deficiency and the ABCB11 deficiency, had lower GGT levels at the second month of life but higher GGT levels beyond 4 months. In addition, GGT levels in patients with a genetic diagnosis were lower than that in patients without a genetic diagnosis at first several months of life.

**Table 4:** GGT levels in patients with or without a genetic diagnosis and controls (median [P25, P75], U/L).

Age	Controls	P _without_	P _ATP8B1_	P _ABCB11_	*X*^*2*^	*P*
2^nd^	86.0[48.7, 123.0]	56.2[44.9, 69.1] *	34.4[21.1, 50.8] *^	42.0[36.0, 50.0] *^	24.302	0.000
3^rd^	43.5.0[29.7, 65.0]	55.3[37.9, 67.8]	36.0[29.3, 49.1] ^	38.0[32.5, 51.5] ^	9.160	0.027
4^th^	32.0[24.0, 43.0]	50.2[32.0, 78.8] *	32.0[22.0, 42.5] ^	33.3[22.0, 40.4] ^	17.501	0.001
5^th^	16.5[12.0, 25.0]	46.5[30.4, 77.9] *	28.7[20.0, 30.0] *^	36.0[24.4, 41.8] *^	45.240	0.000
6^th^	20.0[14.0, 24.2]	38.4[22.6, 64.0] *	22.0[14.0, 40.0] ^	30.2[19.0, 36.8] *	25.812	0.000
7^th^	13.5[10.0, 18.3]	32.5[21.0, 70.0] *	25.4[15.0, 37.3] *	32.0[15.5, 37.0] *	31.922	0.000
8^th^	14.0[10.8, 18.0]	22.0[15.5, 28.9] *	20.0[13.5, 24.0] *	22.0[16.0, 30.0] *	18.882	0.000
9^th^	12.0[10.0, 17.0]	19.0[14.0, 29.0] *	21.0[12.0, 23.0] *	31.9[18.0, 37.0] *	22.544	0.000
10^th^	11.0[9.0, 14.0]	18.0[11.8, 38.9] *	19.3[13.0, 24.0] *	35.0[18.0, 41.3] *	17.133	0.001
11^th^	10.5[8.8, 15.3]	25.0[11.1, 43.7] *	17.0[10.0, 28.3] *	31.0[24.0, 35.0] *	23.085	0.000
12^th^	10.0[8.0, 13.3]	15.0[12.5, 27.0] *	20.0[16.8, 21.0] *	29.5[20.0, 32.0] *^	33.072	0.000
13^th^ ~	10.0[8.0, 14.0]	25.0[14.0, 49.0] *	14.8[13.0, 22.0] *^	21.0[15.0, 28.0] *^	77.903	0.000

P_ATP8B1_, patients with ATP8B1 deficiency; P_ABCB11_, patients with ABCB11 deficiency;. P_without_, patients without a genetic diagnosis.

* compared with controls, *P*<0.05;

^ compared with P _without_, *P*<0.05.

### GGT levels varied with age in patients with a genetic diagnosis

**Fig 1:**
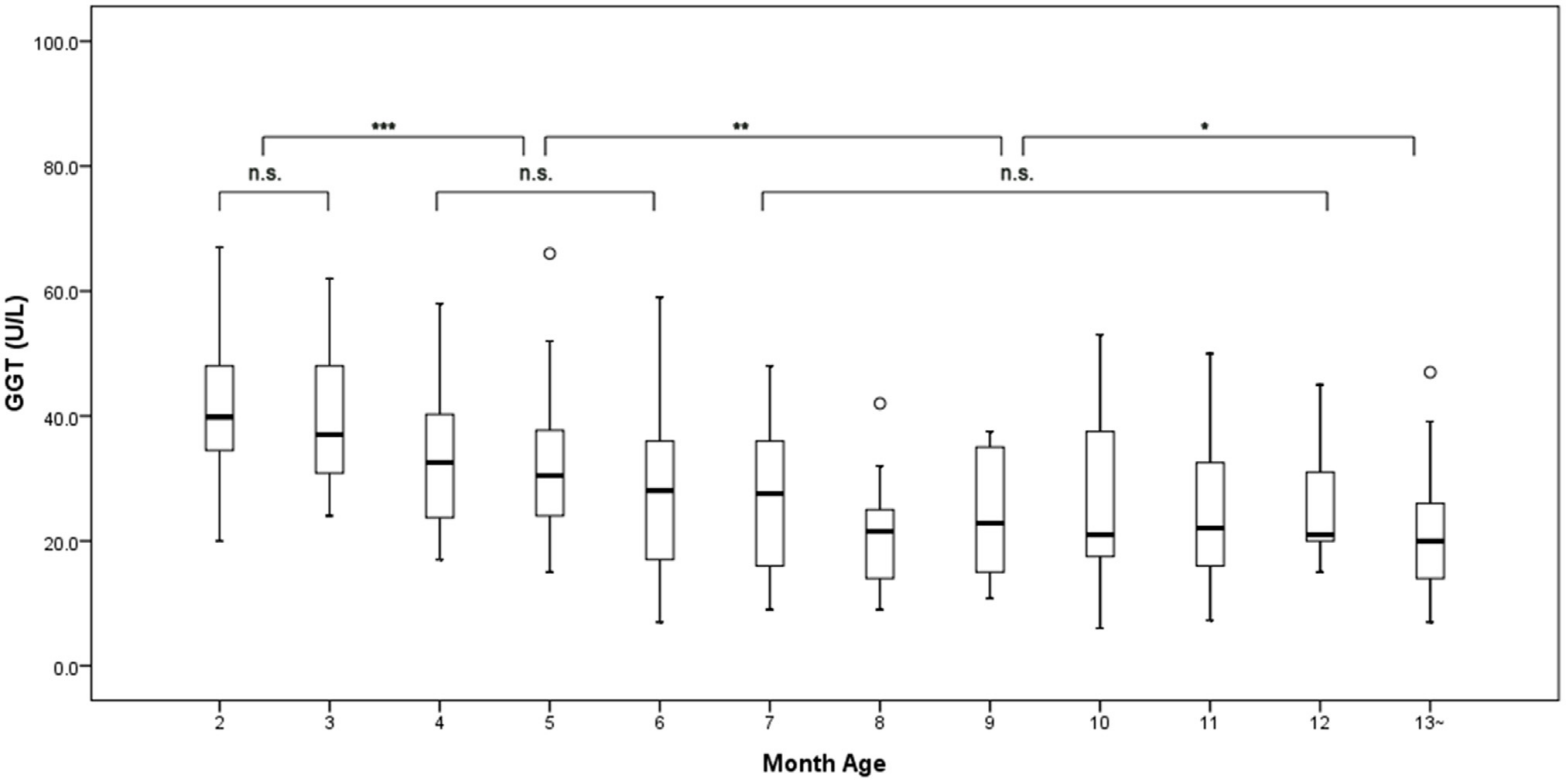
The variation of GGT levels in patients with ATP8B1 or ABCB11 deficiency. *P<0.05; **P<0.01; ***P<0.001; n.s., not significant.

Significant difference was observed when GGT levels were compared among different ages by month in patients with a genetic diagnosis (*X*^*2*^ = 78.430, *P* = 0.000; Fig 1). GGT levels in the 2^nd^ month of life were similar to that in the 3^rd^ month. But GGT levels in the 2^nd^~3^rd^ month were higher than that in the 4^th^~6^th^ month, while they were similar among the 4^th^~6^th^ month. When GGT levels were compared among the 7^th^~12^th^ month, the difference was not significant. GGT levels in the 7^th^~12^th^ month were higher than that beyond 1 year, but were lower than that in the 2^nd^~3^rd^ month and the 4^th^~6^th^ month.

### The ranges for GGT in patients with a genetic diagnosis

Similar ranges for GGT were found in patients with a genetic diagnosis in the 2^nd^~3^rd^ month and the 4^th^~6^th^ month (Table 5). Serum GGT activity was tested 182 times in 36 patients with a genetic diagnosis in the 2^nd^~6^th^ month, the peak GGT value was lower than 70U/L (range: 7.0~67.0U/L). But relatively larger ranges were found in patients without a genetic diagnosis and controls. Serum GGT activities in controls reached adult range (<50U/L) in the 7^th^ month of life. However, one patient with ATP8B1 deficiency and two patients with ABCB11 deficiency had GGT≥50U/L in the 7^th^~12^th^ month. Serum GGT activities of 117 tests revealed that the peak GGT value in patients with a genetic diagnosis was lower than 60U/L in the 7^th^~12^th^ month. All patients with a genetic diagnosis had GGT<50U/L beyond one year, while 37.2% of patients lacking a genetic diagnosis still had GGT≥50U/L.

**Table 5:** The ranges for GGT in patients with or without a genetic diagnosis and controls.

Age	P _with_		P _without_		Controls	
	n ^‡^	GGT (min-max)	n ^‡^	GGT (min-max)	n ^‡^	GGT (min-max)
In the 1^st^ year	299	5.0–67.0U/L	613	5.0–434.2U/L	550	2.0–273.0U/L
2^nd^-3^rd^ month	76	12.0–67.0U/L	236	15.0–434.2U/L	100	15.0–273.0U/L
4^th^-6^th^ month	106	7.0–66.0U/L	199	9.0–364.0U/L	150	4.0–100.0U/L
7^th^-12^th^ month	117	5.0–58.0U/L	178	5.0–257.0U/L	300	2.0–41.0U/L
>1 year old	123	5.3–47.0U/L	155	8.0–356.0U/L	50	4.0–22.0U/L

P_with_, patients with a genetic diagnosis; P_without_, patients without a genetic diagnosis.

^‡^ n, the times of serum GGT activities tests.

### The ranges for GGT and the diagnostic efficiency

39 of the 207 patients, who were ordered to sequence *ATP8B1* and/or *ABCB11* between January 2012 and December 2015, received a molecular diagnosis. The overall rate of a positive molecular diagnosis was 18.8%. GGT levels of 111 (53.6%) patients were found to meet the ranges described in patients with a genetic diagnosis. All the 39 patients with ATP8B1 or ABCB11 deficiency were included in the 111 patients. The sensitivity was 100.0%. The rate of a positive molecular diagnosis increased to 35.1% (35.1% vs. 18.8%, *X*^*2*^ = 10.363, *P* = 0.001). 67 (60.4%) of the 111 patients were ordered to test for both genes, 21 (31.3%) obtained a molecular diagnosis. The 21 patients included 3 patients whose causal gene was identified when the second gene was tested. 44 of the 111 patients were ordered to test for *ATP8B1* or *ABCB11* only, 18 (40.9%) received a molecular diagnosis (18/44 vs. 21/67, *X*^*2*^ = 1.066, *P =* 0.301). GGT activities of the remaining 96 (57.1% of subjects lacking a genetic diagnosis and 46.4% of the 207 patients) patients exceeded the ranges described above. These patients failed to fulfill the definition of ATP8B1 or *ABCB11* deficiency, and accounted for 43.8% of sequencing cost (Table 6).

**Table 6:** Information of genetic tests and sequencing cost.

	Within the ranges ^§^		Over the ranges ^§^	
	Case	Cost ^‡^	Case	Cost ^‡^
Patients with diagnoses	39	60T	0	0
Test for both genes	21	42T	0	0
Test for one gene	18	18T	0	0
Patients without diagnoses	72	118T	96	139T
Test for both genes	46	92T	43	86T
Test for one gene	26	26T	53	53T

^§^ the ranges, the ranges described in patients with ATP8B1 or ABCB11 deficiency. The peak GGT value was <70U/L in the 2^nd^~6^th^ month, <60U/L in the 7^th^~12^th^ month and <50U/L beyond one year.

^‡^ Cost, the sequencing cost. The sequencing cost of *ATP8B1* was equal to that of *ABCB11*. T was defined as the sequencing cost of one gene.

## Discussion

ATP8B1 deficiency and ABCB11 deficiency are the most common causes of chronic cholestasis with low serum GGT activity. They were widely reported around the world including mainland China and Taiwan [16,18–20]. Nevertheless, the ranges for GGT in these patients were unclear. In this study, we found that GGT levels and ranges in patients with ATP8B1 or ABCB11 deficiency were different from that in patients without a genetic diagnosis and controls. GGT levels in these patients varied with age. The peak GGT value was <70U/L in the 2^nd^~6^th^ month, <60U/L in the 7^th^~12^th^ month and <50U/L beyond one year. These features of GGT could greatly improve the diagnostic efficiency.

Mutations in *ATP8B1* or *ABCB11* could result in dysfunction of BSEP, and impaired BA secretion [11]. The reduced bile acids in bile preserved GGT localisation at the canalicular membrane, and resulted in a phenotype with low GGT [10]. GGT levels were found to be similar at presentation or at time of diagnosis between the ATP8B1 deficiency and the ABCB11 deficiency [11,15]. We found that GGT levels in these patients were similar at any observed month of age. It was possible that mutations in *ATP8B1* and *ABCB11* had similar effect on BA secretion. However, higher serum BA and more vitamin deficiency were found in patients with ABCB11 deficiency [11,15]. This indicated that more severe impairment on BA secretion was caused by ABCB11 defect than ATP8B1 defect. It was found that GGT expression at canalicular membrane was more severely impaired in ATP8B1 deficiency patients than ABCB11 deficiency patients [24–25]. Therefore, the impairment on GGT expression and BA secretion might account for the similar GGT levels found in patients with ATP8B1 or ABCB11 deficiency.

Similar to healthy infants [11–12], GGT levels in patients with ATP8B1 or ABCB11 deficiency also varied with age. This might attribute to that GGT expression continued to decline until about 6 months old [13]. However, GGT levels in these patients were different from that in patients without a genetic diagnosis and controls. Larger ranges were found in patients without a genetic diagnosis. The peak GGT value in patients with a genetic diagnosis was lower than 70U/L (1.4 times of the normal adult range) in the 2^nd^~6^th^ month of life. It was lower than that in healthy infants reported previously [12–13] and controls reported in this study. Some controls had GGT≥70U/L in the 2^nd^~6^th^ month, especially at the second month of life. Three patients with a genetic diagnosis had GGT≥50U/L at the 7^th^~12^th^ month of life, while controls reached the adult range (GGT<50U/L) at 6 months old. Therefore, we concluded that GGT<70U/L in the 2^nd^~6^th^ month, <60U/L in the 7^th^~12^th^ month and <50U/L beyond one year were important features of patients with ATP8B1 or ABCB11 deficiency.

It was known that ATP8B1 deficiency and ABCB11 deficiency accounted for the majority of patients with low GGT. These explained why 60.4% of patients whose GGT levels met the ranges described above were ordered to test for both *ATP8B1* and *ABCB11*. However, the rates of a positive molecular diagnosis were similar between patients testing for both genes and patients testing for one gene (*P =* 0.301). This might attribute to clinic and laboratory differences between the two disorders as reported previously [11,15]. We proposed that genetic tests should be ordered according to the differences. As some patients obtained a molecular diagnosis from the second gene test, the second gene should be ordered if the first test revealed negativity. However, other genetic defects (eg, TJP2 [26]) also caused cholestasis with low GGT. Multi-gene panel [27] or whole exons sequencing [28] might be more effective for these patients.

In fact, the ranges for GGT in patients with ATP8B1 or ABCB11 deficiency were unclear when genetic tests were ordered. This caused some patients with elevated GGT were ordered to test for the two genes and resulted in a low rate of positive molecular diagnosis. According to our data, only 18.8% of patients who tested for *ATP8B1* and/or *ABCB11* could be assigned to a molecular diagnosis. 46.4% of patients had GGT activities over the ranges described above. Of these patients, other genetic defects should be taken into consideration. If these patients were waived from testing for these genes, the rate of positive molecular diagnosis would increase greatly. Meanwhile, 43.8% of the sequencing cost would have been saved.

## Conclusion

GGT levels in the ATP8B1 deficiency were similar to that in the ABCB11 deficiency at any observed month of age. Similar to healthy infants, GGT levels in these patients also varied with age. But GGT levels in these patients were different from that in patients without a genetic diagnosis and controls. The peak GGT value was <70U/L in the 2^nd^~6^th^ month, <60U/L in the 7^th^~12^th^ month and <50U/L beyond one year of age. If only patients whose GGT levels met the ranges were ordered to test for these genes, the overall rate of a positive molecular diagnosis would have increased greatly. This also resulted in approximately 1/2 of sequencing cost saving.

## Supporting Information

S1 Checklist STROBE checklist. (DOC)

S1 Table Effect prediction of novel missense mutations. (DOC)
