# Supplementary material for: The Features of GGT in Patients with ATP8B1 or ABCB11 Deficiency Improve the Diagnostic Efficiency
Source: PLoS One. 2016 Apr 6;11(4):e0153114. doi: 10.1371/journal.pone.0153114 (PMC4822785; doi:10.1371/journal.pone.0153114)
Supplement: S1 Checklist — (DOC) [file pone.0153114.s001.doc]

STROBE Statement—checklist of items that should be included in reports of observational studies

|  | Item No. | Recommendation | Page  No. | Relevant text from manuscript |
| --- | --- | --- | --- | --- |
| **Title and abstract** | 1 | (*a*) Indicate the study’s design with a commonly used term in the title or the abstract | 1 | retrospectively |
|  |  | (*b*) Provide in the abstract an informative and balanced summary of what was done and what was found | 1-2 | unravel the features of GGT  GGT levels varied with age |
| Introduction | | | |  |
| Background/rationale | 2 | Explain the scientific background and rationale for the investigation being reported | 3 | The ambiguity of the ranges for GGT might hinder the diagnosis |
| Objectives | 3 | State specific objectives, including any prespecified hypotheses | 3 | unravel the features of GGT |
| Methods | | | |  |
| Study design | 4 | Present key elements of study design early in the paper | N/A | no |
| Setting | 5 | Describe the setting, locations, and relevant dates, including periods of recruitment, exposure, follow-up, and data collection | 3, 5 | 207 patients between January 2012 and December 2015  17 patients with ATPB81 or ABCB11 deficiency diagnosed between January 2004 and December 2011  clinical data were obtained by reviewing medical records |
| Participants | 6 | (*a*) *Cohort study*—Give the eligibility criteria, and the sources and methods of selection of participants. Describe methods of follow-up  *Case-control study*—Give the eligibility criteria, and the sources and methods of case ascertainment and control selection. Give the rationale for the choice of cases and controls  *Cross-sectional study*—Give the eligibility criteria, and the sources and methods of selection of participants | 3 | Patients were ordered to screen for mutations in ATP8B1 and/or ABCB11 for chronic cholestasis  other causes of chronic cholestasis were excluded |
|  |  | (*b*) *Cohort study*—For matched studies, give matching criteria and number of exposed and unexposed  *Case-control study*—For matched studies, give matching criteria and the number of controls per case | 4 | 600 population-matched children severed as controls |
| Variables | 7 | Clearly define all outcomes, exposures, predictors, potential confounders, and effect modifiers. Give diagnostic criteria, if applicable | 4-5 | Subjects with both alleles of ATP8B1 or ABCB11  heterozygote with decreased GGT/BSEP expression |
| Data sources/ measurement | 8* | For each variable of interest, give sources of data and details of methods of assessment (measurement). Describe comparability of assessment methods if there is more than one group | 4 | Genetic analysis and IHC was performed as reported previously |
| Bias | 9 | Describe any efforts to address potential sources of bias | 4 | The pathologists were blinded to genotyping result |
| Study size | 10 | Explain how the study size was arrived at | 3 | 207 patients between January 2012 and December 2015 were enrolled in this study  To expand the patient number, 17 patients with ATPB81 or ABCB11 deficiency diagnosed between January 2004 and December 2011 were also enrolled in this study |

Continued on next page

| Quantitative variables | 11 | Explain how quantitative variables were handled in the analyses. If applicable, describe which groupings were chosen and why | 5 | GGT activities during disease course were used for statistical analysis, but those after liver transplantation, partial billiary diversion or liver failure were discarded. If GGT activities were tested several times within one month, the mean was used for comparison of GGT levels. |
| --- | --- | --- | --- | --- |
| Statistical methods | 12 | (*a*) Describe all statistical methods, including those used to control for confounding | 5-6 | Data were expressed as median [P25, P75] for non-normality. Comparison of GGT levels among three or more groups was performed by the nonparametric Kruskal-Wallis H test. The difference between two ratios was tested by Chi-square test. |
|  |  | (*b*) Describe any methods used to examine subgroups and interactions | 5 | Comparison of GGT levels between two groups was done by the nonparametric Mann-Whitney test. |
|  |  | (*c*) Explain how missing data were addressed | N/A | no |
|  |  | (*d*) *Cohort study*—If applicable, explain how loss to follow-up was addressed  *Case-control study*—If applicable, explain how matching of cases and controls was addressed  *Cross-sectional study*—If applicable, describe analytical methods taking account of sampling strategy | N/A | no |
|  |  | (*e*) Describe any sensitivity analyses | N/A | no |
| Results | | | | |
| Participants | 13* | (a) Report numbers of individuals at each stage of study—eg numbers potentially eligible, examined for eligibility, confirmed eligible, included in the study, completing follow-up, and analysed | 14 and Table 5 | The ranges for GGT in patients with a genetic diagnosis |
|  |  | (b) Give reasons for non-participation at each stage | N/A | no |
|  |  | (c) Consider use of a flow diagram | N/A | no |
| Descriptive data | 14* | (a) Give characteristics of study participants (eg demographic, clinical, social) and information on exposures and potential confounders | 5-6 and Table 1, 2 | Molecular results |
|  |  | (b) Indicate number of participants with missing data for each variable of interest | N/A | no |
|  |  | (c) *Cohort study*—Summarise follow-up time (eg, average and total amount) | N/A | no |
| Outcome data | 15* | *Cohort study*—Report numbers of outcome events or summary measures over time | N/A | no |
|  |  | *Case-control study—*Report numbers in each exposure category, or summary measures of exposure | N/A | no |
|  |  | *Cross-sectional study—*Report numbers of outcome events or summary measures | N/A | no |
| Main results | 16 | (*a*) Give unadjusted estimates and, if applicable, confounder-adjusted estimates and their precision (eg, 95% confidence interval). Make clear which confounders were adjusted for and why they were included | 10 | no significance difference was found when the rates of CMV infection were compared between patients with and without a genetic diagnosis |
|  |  | (*b*) Report category boundaries when continuous variables were categorized | 14 | The ranges for GGT in patients with a genetic diagnosis |
|  |  | (*c*) If relevant, consider translating estimates of relative risk into absolute risk for a meaningful time period | N/A | no |

Continued on next page

| Other analyses | 17 | Report other analyses done—eg analyses of subgroups and interactions, and sensitivity analyses | 15 | The ranges for GGT and the diagnostic efficiency |
| --- | --- | --- | --- | --- |
| Discussion | | | | |
| Key results | 18 | Summarise key results with reference to study objectives | 16 | The peak GGT value was <70U/L in the 2nd~6th month, <60U/L in the 7th~12th month and <50U/L beyond one year |
| Limitations | 19 | Discuss limitations of the study, taking into account sources of potential bias or imprecision. Discuss both direction and magnitude of any potential bias | N/A | no |
| Interpretation | 20 | Give a cautious overall interpretation of results considering objectives, limitations, multiplicity of analyses, results from similar studies, and other relevant evidence | 16-17 | GGT levels in patients with a genetic diagnosis were different from that in patients without a genetic diagnosis and controls. |
| Generalisability | 21 | Discuss the generalisability (external validity) of the study results | 18 | Resulted in approximate 1/2 of sequencing cost saving |
| Other information | |  | | |
| Funding | 22 | Give the source of funding and the role of the funders for the present study and, if applicable, for the original study on which the present article is based | 18 | The National Natural Science Foundation of China (No. 81070281, NO. 81361128006). |

*Give information separately for cases and controls in case-control studies and, if applicable, for exposed and unexposed groups in cohort and cross-sectional studies.

**Note:** An Explanation and Elaboration article discusses each checklist item and gives methodological background and published examples of transparent reporting. The STROBE checklist is best used in conjunction with this article (freely available on the Web sites of PLoS Medicine at http://www.plosmedicine.org/, Annals of Internal Medicine at http://www.annals.org/, and Epidemiology at http://www.epidem.com/). Information on the STROBE Initiative is available at www.strobe-statement.org.
