# Supplementary material for: The Features of GGT in Patients with ATP8B1 or ABCB11 Deficiency Improve the Diagnostic Efficiency
Source: PLoS One. 2016 Apr 6;11(4):e0153114. doi: 10.1371/journal.pone.0153114 (PMC4822785; doi:10.1371/journal.pone.0153114)
Supplement: S1 Table — (DOC) [file pone.0153114.s002.doc]

**S1 Table. Effect Prediction of Novel Missense Mutations**

| Mutation | Mutation Taster | | Polyphen-2 | |
| --- | --- | --- | --- | --- |
|  | Prediction | *P* Value | Prediction | Score |
| Mutation in *ATP8B1* | | | | |
| c.602G>A, p.R201H | disease causing | 0.999 | probably damaging | 1.000 |
| c.1264G>C, p.D422H | disease causing | 0.999 | probably damaging | 1.000 |
| c.2734G>A, p.G912R | disease causing | 0.999 | probably damaging | 1.000 |
| c.1661A>C, p.D554A | disease causing | 0.999 | probably damaging | 1.000 |
| c.1741G>A, p.E581K | disease causing | 0.999 | probably damaging | 0.999 |
| Mutations in *ABCB11* | | | | |
| c.2474A>G, p.E825G | disease causing | 0.999 | probably damaging | 0.995 |
| c.1847G>A, p.R616H | disease causing | 0.999 | probably damaging | 1.000 |
| c.2702G>T, p.S901I | disease causing | 0.999 | possibly damaging | 0.609 |
| c.229C>G, p.P77A | disease causing | 0.999 | possibly damaging | 0.845 |
| c.2542G>A, p.D848N | disease causing | 0.999 | probably damaging | 1.000 |
| c.197G>A, p.S66N | disease causing | 0.998 | possibly damaging | 0.724 |
| c.555G>A, p.M185I | disease causing | 0.999 | possibly damaging | 0.900 |
| c.1762G>C, p.A588P | disease causing | 0.999 | probably damaging | 1.000 |
| c.2603T>A, p.V868D | disease causing | 0.999 | probably damaging | 0.966 |
